# German S3 guideline on implant-supported all-ceramic restorations

**DOI:** 10.1186/s40729-025-00641-7

**Published:** 2025-08-13

**Authors:** Lukas Waltenberger, Shaza Bishti, Stefan Wolfart

**Affiliations:** Department of Prosthodontics and Biomaterials, Center for Implantology, University Hospital RWTH Aachen, Pauwelsstrasse 30, Aachen, 52074 Germany

**Keywords:** All-ceramic, Implant-supported fixed dental prosthesis, FDP, Guideline, Consensus, Crown, Bridge, Full-arch, Evidence-based dentistry, Prosthodontics

## Abstract

**Purpose:**

With increasing demand for aesthetic, metal-free restorations, all-ceramic materials have gained popularity in implant prosthodontics. However, questions regarding their long-term performance, material selection, and design features remain unresolved. This guideline, developed by the German Association of Oral Implantology (DGI) in collaboration with the German Society of Dentistry and Oral Medicine (DGZMK), aims to enhance treatment safety, guide clinical decision-making, and improve patient education concerning all-ceramic implant-supported restorations.

**Methods:**

A systematic literature review was conducted to evaluate the clinical performance of all-ceramic restorations in three main indications: implant-supported single crowns, short-span fixed dental prostheses, and full-arch restorations. Recommendations were developed based on the quality of evidence or expert consensus.

**Results:**

For single crowns, lithium disilicate, silicate ceramics, and all generations of zirconia demonstrated favorable 3-year survival rates (~ 96–97%). In contrast, polymer-infiltrated ceramics showed inferior performance and were not recommended. For short-span FDPs and full-arch reconstructions, only 3Y-TZP zirconia is supported by clinical evidence. Micro-veneering and monolithic designs reduce chipping risks. Patient education is emphasized due to limited evidence for newer materials and full-arch restorations.

**Conclusions:**

All-ceramic implant restorations can be successfully implemented with careful material selection, adherence to bonding protocols, and appropriate prosthetic design. However, clinical limitations persist, especially in full-arch indications. Interdisciplinary collaboration is essential to optimize outcomes and minimize complications.

## Graphical Abstract

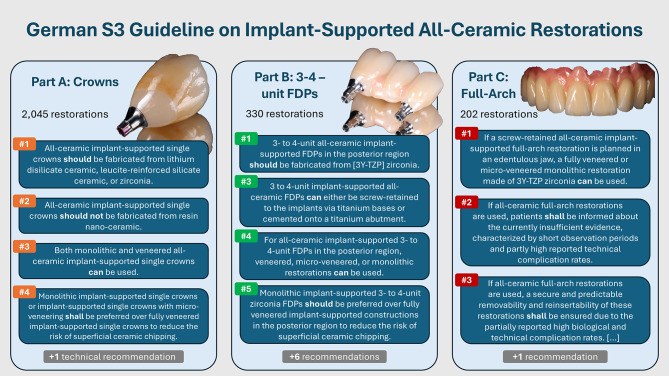

## Background

With the advent of tooth colored, more translucent zirconia ceramics, implant-supported all-ceramic restorations have become a routine solution for replacing missing teeth supported by dental implants. Nevertheless, the specific requirements regarding the material properties of all-ceramic implant-supported restorations remain complex. The introduction of new zirconia generations, which exhibit increased translucency but reduced strength, has brought forth a class of ceramics with specific indication-related limitations. Furthermore, their long-term clinical performance in implant applications has yet to be validated.

This guideline [1] was developed to increase treatment safety and, consequently, enhance patient satisfaction. Additionally, it seeks to improve patient education by highlighting indications with limited scientific evidence. The initiative was led by the German Association of Oral Implantology (DGI) in cooperation with the German Society of Dentistry and Oral Medicine (DGZMK). Three central questions were defined at the outset of the guideline and subsequently addressed:

1. What is the long-term performance of all-ceramic restorations on implants?
2. Which ceramic materials can be recommended for which indication?
3. Which design features can improve the long-term success of all-ceramic restorations on implants?

## Methods

This guideline was developed based on the regulatory of the Association of the Scientific Medical Societies in Germany (AWMF) in accordance with the Appraisal of Guidelines for Research and Evaluation (AGREE II). To address the formulated questions, the literature was systematically reviewed by the authors. The selection and data extraction followed predefined criteria. The quality of evidence was rated with the GRADE approach [2]. In addition to a large body of literature on implant-supported single crowns, only studies on all-ceramic implant-supported restorations with three- to four-unit spans and full-arch restorations could be identified. Consequently, the guideline was divided into three sections to address the available evidence and formulate recommendations for different indications precisely. Recommendations were formulated after group discussion based on evidence or expert consensus. A strong consensus was reached for every recommendation. The strength of the recommendation itself reflects the degree of confidence in the recommendation considering both the quality of evidence and the potential benefits and risks of following it. A recommendation can either be strong (A ⇑⇑, “shall”), moderate (B ⇑, “should”) or weak (0 ⇔, “can”).

## Results

### Part A – Implant-Supported single crowns

Recommendations for all-ceramic implant-supported single crowns are partly based on the 6th Consensus Conference of the European Association of Osseointegration (EAO) [3]. The data pool comprises 2,045 restorations from 52 studies included in a systematic review [4] and additional up to date author searches [5–8]. Table 1 provides an overview of the recommendations outlined in Part A.

Lithium disilicate, (leucite-reinforced) silicate, or zirconia ceramics demonstrate favorable survival rates (96–97% at 3 years) and are recommended for implant-supported single crowns. The recommendation for zirconia is not limited to specific generations. One clinical study successfully evaluated 6Y-PSZ zirconia crowns (6 mol% yttria—partly-stabilized zirconia, ~ 600 MPa flexural strength) over 2 years [7]. By contrast, second-generation tooth-colored opaque zirconia typically shows ~ 1200 MPa flexural strength, with excellent long-term data.

Implant-supported single crowns made of resin nano-ceramic, also referred to as PICN (polymer-infiltrated ceramic network) show variable but consistently lower survival rates, which report as low as 14% after one year [9], and should therefore not be used.

Based on available evidence, the guideline allows flexibility in the extend of veneering implant-supported single crowns. However, monolithic or micro-veneered designs are strongly preferred over fully veneered crowns due to a reduced risk of ceramic chipping. Micro-veneering involves a 0.5 mm layer in non-functional areas. Another recommendation highlights the importance of knowledge about material properties and proper application of intra- and extraoral bonding protocols by both clinicians and dental technicians. For all-ceramic restorations, knowledge and adherence to protocols is critical for success. 

**Table 1:** Recommendations for Implant-Supported All-Ceramic crowns

Recommendations for Implant-Supported All-Ceramic Crowns		
*Recommendations*	*Basis and strength of consensus*	*Recommendation grade*
A1: All-ceramic implant-supported single crowns **should** be fabricated from lithium disilicate ceramic, leucite-reinforced silicate ceramic, or zirconia. *	evidence-based, strong consensus	B ⇑
A2: All-ceramic implant-supported single crowns **should not** be fabricated from resin nano-ceramic.	evidence-based, strong consensus	B ⇑
A3: Both monolithic and veneered all-ceramic implant-supported single crowns **can** be used. *	evidence-based, strong consensus	0 ⇔
A4: Monolithic implant-supported single crowns or implant-supported single crowns with micro-veneering **shall** be preferred over fully veneered implant-supported single crowns to reduce the risk of superficial ceramic chipping.	evidence-based, strong consensus	A ⇑⇑
A5: For a successful outcome with all-ceramic implant-supported single crowns, thorough knowledge of the material and precise application of the recommended protocols for both conventional cementation and adhesive bonding are essential. These protocols **shall** be applied for both extraoral (using titanium bases) and intraoral bonding.	consensus-based, strong consensus	A ⇑⇑

*= No robust evidence is available for monolithic zirconia crowns in the anterior region

### Part B – Short-Span Implant-Supported FDPs

The systematic literature review identified nine clinical studies with a total of 330 short-span implant-supported fixed dental prostheses (i-FDPs) [8, 10–17]. Only zirconia was found suitable and should exclusively be used in this indication. Sufficient data exists only for second-generation 3Y-TZP zirconia with > 1000 MPa flexural strength. There is no clinical data for newer generations or material combinations. An overview of the recommendations from Part B is presented in Table 2.

Thus, a separate recommendation addresses the wide variability of zirconia generations. Patient education regarding the limited data for newer zirconia types (e.g., multilayers) is essential. No general superiority was found for cemented versus screw-retained short-span i-FDPs. Cementation should be performed on customized abutments to allow better control of excess material. Screw-retention should utilize titanium bases with sufficient height and parallel retention surfaces.

As with crowns, micro-veneering reduces chipping risk in short-span i-FDPs. In posterior regions, monolithic or micro-veneered designs are preferred. Some promising clinical data exist for cantilevered all-ceramic i-FDPs, but patient education about limited evidence is necessary [18–20].

**Table 2:** Recommendations for Short-Span Implant-Supported FDPs

Recommendations for Short-Span Implant-Supported FDPs		
*Recommendations*	*Basis and strength of consensus*	*Recommendation grade*
B1: 3- to 4-unit all-ceramic implant-supported FDPs in the posterior region **should** be fabricated from zirconia. The supporting evidence […] is based on studies reporting on 3Y-TZP zirconia with a flexural strength > 1000 MPa.	evidence-based, strong consensus	B ⇑
B2: Different types and generations of zirconia exhibit variations in optical and biomechanical properties. The identified clinical evidence refers exclusively to 3Y-TZP zirconia. For other generations and combinations, no sufficient clinical evidence is currently available.	consensus-based, strong consensus	statement
B3: 3- to 4-unit implant-supported all-ceramic FDPs **can** either be screw-retained to the implants via titanium bases or cemented onto a titanium abutment.	evidence-based, strong consensus	0 ⇔
B4: For all-ceramic implant-supported 3- to 4-unit FDPs in the posterior region, veneered, micro-veneered, or monolithic restorations **can** be used.	evidence-based, strong consensus	0 ⇔
B5: Monolithic implant-supported 3- to 4-unit zirconia FDPs **should** be preferred over fully veneered implant-supported constructions in the posterior region to reduce the risk of superficial ceramic chipping.	evidence-based, strong consensus	B ⇑
B6: In the anterior region, 3- to 4-unit micro-veneered monolithic zirconia FDPs **can** be preferred over fully veneered restorations to reduce the risk of superficial ceramic chipping.	consensus-based, strong consensus	0 ⇔
B7: If 3- to 4-unit implant-supported all-ceramic FDPs are screw-retained, they **should** be connected to the implant using a titanium base.	consensus-based, strong consensus	B ⇑
B8: When using titanium bases, attention **should** be paid to sufficient height, parallel retention surfaces, and extraoral bonding in order to prevent loss of retention.	consensus-based, strong consensus	B ⇑
B9: The cementation or adhesive bonding of all-ceramic implant-supported 3- to 4-unit FDPs **should** be performed on individually fabricated titanium or hybrid abutments to enable safe removal of excess bonding material.	consensus-based, strong consensus	B ⇑
B10: If all-ceramic implant-supported cantilever FDPs are to be used, patients **shall** be informed about the little evidence regarding their long-term clinical performance.	consensus-based, strong consensus	A ⇑⇑

### Part C – Implant-Supported Full-Arch restorations

The body of evidence for full-arch implant-supported all-ceramic restorations stems from the authors’ systematic literature search. It remains limited, with only 202 restorations reported across three clinical studies [21–23]. Clinical data is available exclusively for 3Y-TZP zirconia, which is therefore the only material currently recommended. Table 3 summarizes the recommendations derived from Part C.

Because of the scarcity of data and short observation periods, no recommendation is made regarding veneering design. High complication rates have been reported. Therefore, a strong recommendation is made for thorough patient education when using an all-ceramic full-arch restoration. Given the complication risks, restorations should allow removability and reinsertion, using screw-retained connections via titanium bases or multi-unit abutments. Additionally, protective splints are recommended to prevent complications.

Overall, metal-ceramic restorations remain the gold standard in this indication due to the limited evidence for all-ceramic alternatives.

**Table 3:** Recommendations for Full-Arch Implant-Supported All-Ceramic restorations

Recommendations for Implant-Supported Full-Arch Restorations		
*Recommendations*	*Basis and strength of consensus*	*Recommendation grade*
C1: If a screw-retained all-ceramic implant-supported full-arch restoration is planned in an edentulous jaw, a fully veneered or micro-veneered monolithic restoration made of 3Y-TZP zirconia **can** be used.	evidence-based, strong consensus	0 ⇔
C2: If all-ceramic full-arch restorations are used, patients **shall** be informed about the currently insufficient evidence, characterized by short observation periods and partly high reported technical complication rates.	evidence-based, strong consensus	A ⇑⇑
C3: If all-ceramic full-arch restorations are used, a secure and predictable removability and reinsertability of these restorations **shall** be ensured due to the partially reported high biological and technical complication rates – achieved through screw retention using titanium bases, angled screw channel systems, or multi-unit abutments.	consensus-based, strong consensus	A ⇑⇑
C4: Due to the complete absence of periodontal receptors in purely implant-supported all-ceramic full-arch restorations, the use of a protective splint **can** be considered to prevent technical complications.	consensus-based, strong consensus	0 ⇔

### Part D – General considerations

Part D presents overarching recommendations for all-ceramic implant-supported restorations. In cases of diagnosed or suspected bruxism, patients should be informed about the increased risk of complications, the need for a nocturnal protective splint, and possible manufacturer-imposed indication limits. All-ceramic restorations adjusted intraorally should be polished to high gloss to ensure surface integrity. Clinicians should consider material-specific biomechanical risks and perform regular occlusal checks, especially for monolithic zirconia, to avoid overloading the implant–abutment connection. Given the wide range of ceramic materials and compositions, particularly varying zirconia generations, material selection should be coordinated between clinician and dental technician.

## Conclusions

This guideline provided structured, evidence- and consensus-based recommendations for the clinical use and design of all-ceramic implant-supported single crowns, three- to four-unit implant-supported FDPs and full-arch restorations. While the evidence for single crowns and short-span FDPs was sufficiently robust to formulate comprehensive recommendations, evidence for full-arch restorations remained limited. Therefore, the guideline emphasized thorough patient education about missing long-term data and high complication rates in the provision of all-ceramic full-arch restorations. Due to the diversity of ceramic materials and the evolving landscape of zirconia generations, close interdisciplinary collaboration between clinicians and dental technicians is crucial to ensure indication-appropriate and long-term successful restorations.

## Data Availability

The material and data that support the findings of this article are available publicly in the methodology of this guideline. See: https://register.awmf.org/assets/guidelines/083-053m_S3_Vollkeramische-festsitzende-implantatgetragene-Restaurationen_2025-02.pdf. The guideline can be assessed at: https://register.awmf.org/assets/guidelines/083-053l_S3_Vollkeramische-festsitzende-implantatgetragene-Restaurationen_2024-12.pdf

## Abbreviations

FDP: fixed dental prosthesis
i-FDP: implant-supported fixed dental prosthesis
3Y-TZP: 3 mol% yttria-stabilized zirconia
6Y-PSZ: 6 mol% yttria-partly stabilized zirconia
MPa: megapascal

## References

[1] Waltenberger L, Wolfart S. In: DGI D, editor. Vollkeramische festsitzende implantatgetragene restaurationen. Germany: AWMF; 2024. p. 117.

[2] Balshem H, Helfand M, Schunemann HJ, Oxman AD, Kunz R, Brozek J, et al. GRADE guidelines: 3. Rating the quality of evidence. J Clin Epidemiol. 2011;64(4):401–6. 21208779 10.1016/j.jclinepi.2010.07.015

[3] Jokstad A, Pjetursson BE, Mühlemann S, Wismeijer D, Wolfart S, Fehmer V et al. Fabrication, workflow and delivery of reconstruction: Summary and consensus statements of group 4. The 6th EAO Consensus Conference 2021. Clin Oral Implants Res. 2021;32 Suppl 21:336– 41. 10.1111/clr.13797 34145922

[4] Pjetursson BE, Sailer I, Latyshev A, Rabel K, Kohal RJ, Karasan D. A systematic review and meta-analysis evaluating the survival, the failure, and the complication rates of veneered and monolithic all-ceramic implant-supported single crowns. Clin Oral Implants Res. 2021;32(Suppl 21):254–88. 34642991 10.1111/clr.13863 PMC9293296

[5] Zhang Y, Wei D, Tian J, Zhao Y, Lin Y, Di P. Clinical evaluation and quantitative occlusal change analysis of posterior implant-supported all-ceramic crowns: A 3-year randomized controlled clinical trial. Clin Oral Implants Res. 2023;34(11):1188–97. 37526213 10.1111/clr.14151

[6] Wolfart S, Rittich A, Gross K, Hartkamp O, von der Stuck A, Raith S, et al. Cemented versus screw-retained posterior implant-supported single crowns: A 24-month randomized controlled clinical trial. Clin Oral Implants Res. 2021;32(12):1484–95. 34547824 10.1111/clr.13849

[7] Salem MT, El-Layeh M, El-Farag SAA, Salem AS, Attia A. Clinical assessment of different implant-supported esthetic crown systems fabricated with semi-digital workflow: Two-year prospective study. J Esthet Restor Dent. 2022;34(8):1247–62. 36120840 10.1111/jerd.12961

[8] Derksen W, Wismeijer D. Three-Year Follow-up of a randomized clinical trial on Screw-Retained monolithic zirconia restorations on Ti-Base abutments based on digital or conventional impression techniques. Int J Prosthodont. 2023;36(4):410–5. 37699181 10.11607/ijp.7891

[9] Schepke U, Meijer HJ, Vermeulen KM, Raghoebar GM, Cune MS. Clinical bonding of resin nano ceramic restorations to zirconia abutments: A case series within a randomized clinical trial. Clin Implant Dent Relat Res. 2016;18(5):984–92. 26456161 10.1111/cid.12382

[10] Spies BC, Witkowski S, Vach K, Kohal RJ. Clinical and patient-reported outcomes of zirconia-based implant fixed dental prostheses: results of a prospective case series 5 years after implant placement. Clin Oral Implants Res. 2018;29(1):91–9. 28940708 10.1111/clr.13072

[11] Pol CW, Raghoebar GM, Cune MS, Meijer HJ. Implant-Supported Three-Unit fixed dental prosthesis using coded healing abutments and fabricated using a digital workflow: A 1-Year prospective case series study. Int J Prosthodont. 2020;33(6):609–19. 33284902 10.11607/ijp.6707

[12] Koenig V, Wulfman C, Bekaert S, Dupont N, Le Goff S, Eldafrawy M, et al. Clinical behavior of second-generation zirconia monolithic posterior restorations: Two-year results of a prospective study with ex vivo analyses including patients with clinical signs of Bruxism. J Dent. 2019;91:103229. 31722238 10.1016/j.jdent.2019.103229

[13] Ferrini F, Capparé P, Vinci R, Gherlone EF, Sannino G. Digital versus traditional workflow for posterior maxillary rehabilitations supported by one straight and one Tilted implant: A 3-Year prospective comparative study. Biomed Res Int. 2018;2018:4149107. 30534562 10.1155/2018/4149107 PMC6252190

[14] Esquivel-Upshaw JF, Mecholsky JJ Jr., Clark AE, Jenkins R, Hsu SM, Neal D, et al. Factors influencing the survival of implant-supported ceramic-ceramic prostheses: A randomized, controlled clinical trial. J Dent. 2020;103s:100017. 34059304 10.1016/j.jjodo.2020.100017 PMC9993352

[15] Degidi M, Nardi D, Sighinolfi G, Degidi D, Piattelli A. Fixed partial restorations made of a new Zirconia-Reinforced lithium silicate material: A 2-Year Short-Term report. Int J Prosthodont. 2021;34(1):37–46. 33570518 10.11607/ijp.6924

[16] Degidi M, Nardi D, Gianluca S, Piattelli A. The conometric concept: A 5-Year Follow-up of fixed partial monolithic zirconia restorations supported by Cone-in-Cone abutments. Int J Periodontics Restor Dent. 2018;38(3):363–71. 10.11607/prd.3130 29641625

[17] Cheng CW, Chien CH, Chen CJ, Papaspyridakos P. Clinical results and technical complications of posterior Implant-Supported modified monolithic zirconia single crowns and Short-Span fixed dental prostheses: A 2-Year pilot study. J Prosthodont. 2018;27(2):108–14. 29086467 10.1111/jopr.12682

[18] Van Nimwegen WG, Raghoebar GM, Tymstra N, Vissink A, Meijer HJA. How to treat two adjacent missing teeth with dental implants. A systematic review on single implant-supported two-unit cantilever fdp’s and results of a 5-year prospective comparative study in the aesthetic zone. J Oral Rehabil. 2017;44(6):461–71. 28301683 10.1111/joor.12507

[19] Roccuzzo A, Fanti R, Mancini L, Imber JC, Stahli A, Molinero-Mourelle P, et al. Implant-supported fixed dental prostheses with cantilever extensions: state of the Art and future perspectives. Int J Oral Implantol (Berl). 2023;16(1):13–28. 36861678

[20] Jensen-Louwerse C, Sikma H, Cune MS, Gulje FL, Meijer HJA. Single implant-supported two-unit cantilever fixed partial dentures in the posterior region: a retrospective case series with a mean follow-up of 6.5 years. Int J Implant Dentistry. 2021;7(1):78. 10.1186/s40729-021-00361-8 PMC8374011 34409508

[21] Papaspyridakos P, Lal K. Computer-assisted design/computer-assisted manufacturing zirconia implant fixed complete prostheses: clinical results and technical complications up to 4 years of function. Clin Oral Implants Res. 2013;24(6):659–65. 22413889 10.1111/j.1600-0501.2012.02447.x

[22] Limmer B, Sanders AE, Reside G, Cooper LF. Complications and patient-centered outcomes with an implant-supported monolithic zirconia fixed dental prosthesis: 1 year results. J Prosthodont. 2014;23(4):267–75. 24393461 10.1111/jopr.12110

[23] Caramês J, Marques D, Malta Barbosa J, Moreira A, Crispim P, Chen A. Full-arch implant-supported rehabilitations: A prospective study comparing porcelain-veneered zirconia frameworks to monolithic zirconia. Clin Oral Implants Res. 2019;30(1):68–78. 30521106 10.1111/clr.13393

